# Association between masseter muscle sarcopenia and postoperative pneumonia in patients with esophageal cancer

**DOI:** 10.1038/s41598-022-20967-1

**Published:** 2022-09-30

**Authors:** Teppei Kamada, Hironori Ohdaira, Eisaku Ito, Junji Takahashi, Keigo Nakashima, Yuichi Nakaseko, Norihiko Suzuki, Masashi Yoshida, Ken Eto, Yutaka Suzuki

**Affiliations:** 1 Department of Surgery, International University of Health and Welfare Hospital, 537-3, Iguchi, Nasushiobara, Tochigi 329-2763 Japan; 2 Department of Surgery, The Jikei University School of Medicine, 3-25-8, Nishi-Shimbashi, Minato-Ku, Tokyo, 105-8461 Japan

**Keywords:** Cancer, Gastroenterology, Risk factors

## Abstract

Sarcopenia affects the swallowing and chewing muscles, such as the masseter muscle. However, the significance of masseter muscle loss in pneumonia remains unclear. We investigated the effects of masseter muscle sarcopenia (MMS) on postoperative pneumonia in patients with esophageal cancer. In this retrospective cohort study, we analyzed the data of 86 patients who underwent esophagectomy for stage I–III esophageal cancer at our hospital between March 2013 and October 2021. The primary endpoint was postoperative pneumonia within 3 months of surgery. MMS was defined as a (1) masseter muscle index (MMI) that was less than the sex-specific MMI cutoff values, and (2) sarcopenia diagnosed using the L3-psoas muscle index (L3-PMI). Postoperative pneumonia was noted in 27 (31.3%) patients. In multivariate analysis, FEV_1.0_ < 1.5 L (odds ratio, OR: 10.3; 95% confidence interval, CI 1.56–67.4; p = 0.015), RLNP (OR: 5.14; 95%CI 1.47–17.9; p = 0.010), and MMS (OR: 4.83; 95%CI 1.48–15.8; p = 0.009) were independent risk factors for postoperative pneumonia. The overall survival was significantly worse in patients with pneumonia (log-rank: p = 0.01) than in those without pneumonia. Preoperative MMS may serve as a predictor of postoperative pneumonia in patients with esophageal cancer.

## Introduction

Esophagectomy with three-field lymph node dissection for esophageal cancer is one of the most invasive and high-risk surgical procedures for gastrointestinal surgery^1^. Postoperative pneumonia is the commonest complication, which occurs in 20–40% of patients and results in prolonged postoperative hospital stay and higher mortality^2,3^.

The risk factors for postoperative pneumonia include older age, diabetes mellitus, malnutrition, obesity, open thoracotomy, preoperative respiratory comorbidities, increased intraoperative blood loss, and recurrent laryngeal nerve paralysis (RLNP)^4–7^. Nishigori et al. reported that sarcopenia evaluated using the L3 skeletal muscle mass index (SMI) predicts pulmonary complications following esophagectomy for esophageal cancer^8^.

In contrast, sarcopenia is a whole-body process, which also affects muscles related to mastication and swallowing^9,10^. The masseter muscle is the main masticatory and is a skeletal muscle. The fibers of masticatory muscles develop atrophy due to aging, tooth loss, and denture problems. Therefore, the evaluation of masseter muscle thickness is a useful index of occlusal force and masticatory ability. A decrease in masseter muscle thickness leads to a decrease in masticatory ability, which results in malnutrition and pneumonia^11^. Esophageal cancer most commonly results in malnutrition due to its localization^12^. Therefore, we hypothesized that the masseter muscle loss due to preoperative malnutrition in esophageal cancer could be a risk factor for postoperative pneumonia following esophagectomy. The L3-SMI and L3-psoas muscle index (PMI)^13^ are useful predictors of postoperative pneumonia or poor survival^8^; however, the impact of the masseter muscle loss due to sarcopenia on pneumonia after esophagectomy remains unclear in the field of gastrointestinal surgery. Therefore, the purpose of this study was to evaluate whether the masseter muscle sarcopenia diagnosed by the masseter muscle index (MMI) can be a predictor of postoperative pneumonia in esophageal cancer.

## Results

### Patient characteristics

A total of 86 patients were enrolled in the study. The patients’ baseline characteristics, oncological factors, and perioperative factors are summarized in Table 1. These included 76 men and 10 women, with a median age of 70.3 ± 9.1 years. The histological types were squamous cell carcinoma in 74 (86.0%) patients, adenocarcinoma in 9 (10.4%) patients, and others in 3 (3.6%) patients. Sixty-eight patients (79.1%) had thoracic esophageal cancer (79.1%), 14 had abdominal esophageal cancer (16.2%), and 4 (4.7%) had cervical esophageal cancer. The pathological stages were as follows: stage I, n = 33 (38.4%); stage II, n = 20 (23.3%); and stage III, n = 33 (38.3%). Neoadjuvant chemotherapy was administered to 20 (23.3%) patients, preoperative radiation therapy was administered to 5 (5.8%) patients, and postoperative adjuvant chemotherapy was administered to 56 (65.1%) patients. Thoracoscopic and laparoscopic esophagectomies were performed in 49 (57.0%) patients, hand-assisted laparoscopic surgery (HALS) was performed in 32 (37.2%) patients, and robot-assisted esophagectomy was performed in 5 (5.8%) patients. Postoperative pneumonia was observed in 27 (31.4%) patients, anastomotic leakage was observed in 16 (18.6%) patients, and RLNP was observed in 23 (26.7%) patients. Sarcopenia was diagnosed in 47 (54.6%) patients and MMS was diagnosed in 29 (33.7%) patients. The median L3-PMI was 5.91 (range: 1.49–11.4) cm^2^/m^2^ in men and 3.74 (range: 2.46–6.04) cm^2^/m^2^ in women. The median MMI was 3.48 (range: 1.26–7.70) cm^2^/m^2^ in men and 3.39 (range: 1.70–5.27) cm^2^/m^2^ in women.

**Table 1:** Demographic and clinicopathological characteristics of the patient cohort.

Variable		Total	Pneumonia	Non-pneumonia	Univariate p-value	Multivariate OR (95% CI)
			n (%) or median (range)			
Patients		86	27	59		
Age (years)		70.3 ± 9.1	73.0 ± 8.6	69.1 ± 9.2	0.06	
Sex	Male	76 (88.4%)	22 (81.5%)	54 (91.5%)	0.18	
	Female	10 (11.6%)	5 (18.5%)	5 (8.5%)		
Body mass index (kg/m^2^)		19.1 ± 3.5	19.1 ± 4.9	19.2 ± 2.6	0.23	
**Histopathology**					0.87	
Squamous cell carcinoma		74 (86.0%)	23 (85.2%)	51 (86.4%)		
Adenocarcinoma		9 (10.4%)	2 (7.4%)	7 (11.9%)		
Other		3 (3.6%)	2 (7.4%)	1 (1.7%)		
**Primary tumor location**					0.02	0.123 5.45 (0.63–46.9)
Cervical		4 (4.7%)	3 (11.1%)	1 (1.7%)		
Thoracic		68 (79.1%)	23 (85.2%)	45 (76.3%)		
Abdominal		14 (16.2%)	1 (3.7%)	13 (22.0%)		
**Pathological stage**					0.33	
I		33 (38.4%)	12 (44.4%)	21 (35.6%)		
II		20 (23.3%)	3 (11.1%)	17 (28.8%)		
III		33 (38.3%)	12 (44.5%)	21 (35.6%)		
Preoperative chemotherapy		20 (23.3%)	9 (33.3%)	11 (18.6%)	0.14	
Preoperative radiotherapy		5 (5.8%)	3 (11.1%)	2 (3.4%)	0.16	
Adjuvant chemotherapy		56 (65.1%)	12 (44.4%)	44 (74.6%)	< 0.001	NA
HALS		32 (37.2%)	9 (33.3%)	23 (38.9%)	0.62	
Robot-assisted surgery		5 (5.8%)	2 (7.4%)	3 (5.1%)	0.67	
Operation time, min		457.2 ± 66.2	449.5 ± 58.9	460.7 ± 69.5	0.23	
Intraoperative blood loss, mL		170.3 ± 185.7	210.7 ± 265.6	151.7 ± 133.4	0.67	
COPD		5 (5.8%)	3 (11.1%)	2 (3.4%)	0.16	
Diabetes mellitus		11 (12.8%)	5 (18.5%)	6 (10.2%)	0.28	
Hypertension		28 (32.6%)	10 (37.0%)	18 (30.5%)	0.55	
Coronary artery disease		6 (6.9%)	3 (11.1%)	3 (5.1%)	0.31	
Current smoker		47 (54.7%)	14 (51.9%)	33 (55.9%)	0.72	
%VC < 80%		8 (9.3%)	4 (14.8%)	4 (6.8%)	0.23	
FEV_1.0_ < 1.5 L		8 (9.3%)	6 (22.2%)	2 (3.4%)	0.005	0.015 10.3 (1.56–67.4)
Anastomotic leakage		16 (18.6%)	6 (22.2%)	10 (16.9%)	0.56	
RLNP		23 (26.7%)	11 (40.7%)	12 (20.3%)	0.047	0.010 5.14 (1.47–17.9)
Ileus		6 (6.9%)	3 (11.1%)	3 (5.1%)	0.31	
Sarcopenia		47 (54.6%)	18 (66.7%)	29 (49.2%)	0.13	
Masseter muscle sarcopenia		29 (33.7%)	14 (51.8%)	15 (25.4%)	0.016	0.009 4.83 (1.48–15.8)
Sarcopenia without masseter muscle loss		18 (20.9%)	4 (14.8%)	14 (23.7%)	0.35	
Masseter muscle loss without sarcopenia		14 (16.3%)	3 (11.1%)	11(18.6%)	0.38	
Recurrence		36 (41.9%)	10 (37.0%)	26 (44.1%)	0.54	
GPS ≥ 1		16 (18.6%)	5 (18.5%)	11 (18.6%)	0.98	

Preoperative and perioperative risk factors for pneumonia in patients undergoing esophagectomy.

COPD, chronic obstructive pulmonary disease; %VC, vital capacity; FEV_1.0,_ forced expiratory volume in one second; HALS, hand-assisted laparoscopic surgery; RLNP, recurrent laryngeal nerve paralysis; GPS, Glasgow prognostic score.

Grade II, III, and IV pneumonia were noted in 20 (74.1%), 5 (18.5%), and 2 (7.4%) patients, respectively. Pneumonia was diagnosed in the acute phase in 15 (17.4%) patients and in the subacute phase in 12 (13.9%) patients.

The median follow-up period was 33.0 (1.4–91.3) months. During the follow-up period, 41 (47.6%) patients died and 36 (41.8%) patients relapsed.

All 86 patients were divided into pneumonia and non-pneumonia groups (Table 1). In the univariate analysis, postoperative pneumonia was significantly commoner in patients with cervical or thoracic esophageal cancer (p = 0.02), no adjuvant chemotherapy (p < 0.01), FEV_1_ < 1.5 L (p = 0.005), RLNP (p = 0.047), and MMS (p = 0.016). There were no significant differences in age, Glasgow prognostic score, preoperative chemotherapy, pathological stage, sarcopenia, or other perioperative factors.

All variables that demonstrated a significant difference in univariate analysis were included in the multivariate analysis using a logistic regression model. Adjuvant chemotherapy was excluded from the multivariate analysis because postoperative adjuvant chemotherapy and postoperative pneumonia are confounding factors. In the multivariate analysis, FEV_1_ < 1.5 L (odds ratio [OR]: 10.3; [95% confidence interval [CI]: 1.56–67.4], p = 0.015), RLNP (OR: 5.14; [95%CI 1.47–17.9]; p = 0.010), and MMS (OR: 4.83 [95%CI 1.48–15.8], p = 0.009) were found to be independent risk factors for postoperative pneumonia.

### Subgroup analysis

We conducted an analysis in the subgroups of acute phase and subacute phase pneumonia (Table 2). In the multivariate analysis, FEV_1_ < 1.5 L (OR: 10.5; [95%CI 1.63–67.9]; p = 0.013) and RLNP (OR: 11.2; [95%CI 2.57–48.7]; p = 0.001) were found to be independent risk factors for acute phase pneumonia. Although without statistical significance, MMS tended to be a risk factor for both acute phase pneumonia and subacute phase pneumonia (OR: 3.62; [95%CI 0.87–15.1]; p = 0.077), (OR: 3.13; [95%CI 0.88–11.1]; p = 0.078).

**Table 2:** Subgroup analysis of risk factors for acute phase and subacute phase pneumonia in patients undergoing esophagectomy.

Variable	Acute phase pneumonia		Subacute phase pneumonia	
	HR (95% CI)	p-value	HR (95% CI)	p-value
FEV_1.0_ < 1.5 L	10.5 (1.63–67.9)	0.013	2.29 (0.38–13.7)	0.363
RLNP	11.2 (2.57–48.7)	0.001	0.59 (0.11–3.09)	0.537
Masseter muscle sarcopenia	3.62 (0.87–15.1)	0.077	3.13 (0.88–11.1)	0.078

FEV_1.0,_ forced expiratory volume in one second; RLNP, recurrent laryngeal nerve paralysis.

### Effect of pneumonia on disease-free survival (DFS) and overall survival (OS) after esophagectomy

**Figure 1:**
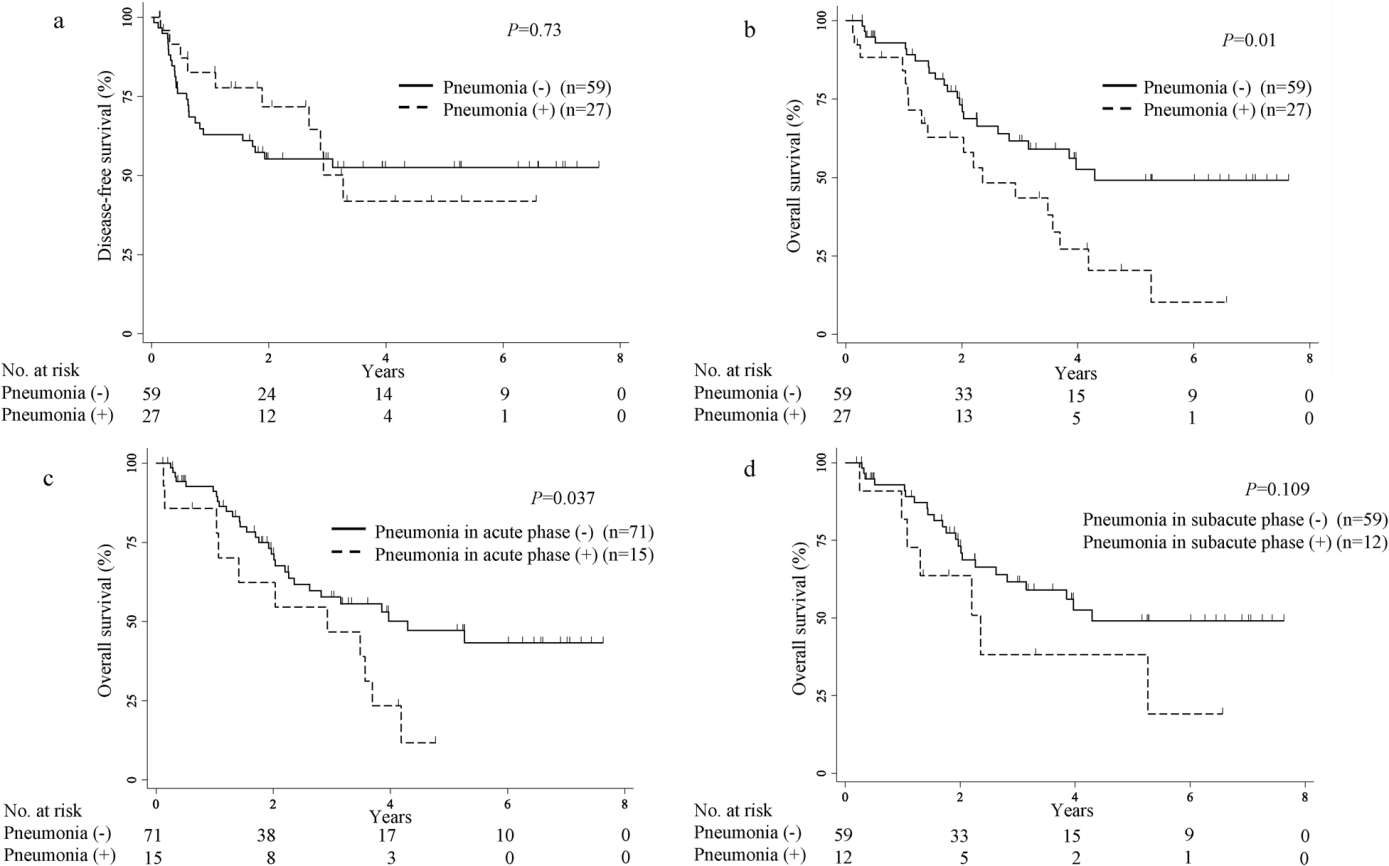
Kaplan–Meier survival curves with log-rank test. (**a**) Comparison of disease-free survival after esophagectomy between patients with pneumonia and those without pneumonia. (**b**) Comparison of overall survival after esophagectomy between patients with pneumonia and those without pneumonia. (**c**) Comparison of overall survival after esophagectomy between patients with pneumonia in the acute phase and those without pneumonia in the acute phase. (**d**) Comparison of overall survival after esophagectomy between patients with pneumonia in the subacute phase and those without pneumonia.

Figure 1 presents the Kaplan–Meier survival curve for DFS and OS. There were no significant differences in the survival rates between the patients with pneumonia and those without pneumonia for DFS (3-year survival, 50.2% [95%CI 24.6–71.2%] vs. 55.2% [95%CI 41.2–67.2%]; p = 0.73, log-rank) (Fig. 1a).

Patients with pneumonia had significantly lower OS than did those without pneumonia (3-year survival, 43.5% [95%CI 22.8–62.4%] vs. 61.6% [95%CI 46.3–73.7%], p = 0.01, log-rank) (Fig. 1b). Furthermore, we conducted a sensitivity analysis in the subgroups of those with pneumonia in the acute phase and those with pneumonia in the subacute phase (Fig. 1c,d).

Patients with pneumonia in the acute phase had significantly lower OS than did those without pneumonia in the acute phase (3-year survival, 46.7% [95%CI 19.6–70.1%] vs. 57.7% [95%CI 43.8–69.3%], p = 0.037, log-rank); however, there were no significant differences in the OS rates between patients with pneumonia in the subacute phase and those without pneumonia (3-year survival, 38.2% [95%CI 9.9–66.9%] vs. 61.6% [46.4–73.7%], p = 0.109, log-rank).

Table 3 summarizes the univariate and multivariate analyses of the association between the clinicopathological characteristics and OS in patients with esophageal cancer. In univariate analysis, the OS rate was significantly worse in patients with stage ≥ II (p < 0.001), acute phase pneumonia (p = 0.042), and masseter muscle sarcopenia (p < 0.001). In multivariate analysis, stage ≥ II (hazards ratio [HR]: 3.82; 95%CI 1.66–8.81; p = 0.002), and masseter muscle sarcopenia (HR: 3.10; 95% CI 1.64–5.86; p = 0.001) were found to be significant independent predictors of OS.

**Table 3:** Univariate and multivariate analysis for overall survival in patients after esophagectomy.

Variable	OS univariate analysis		OS multivariate analysis	
	HR (95% CI)	p-value	HR (95% CI)	p-value
Age, ≥ 65 years	0.88 (0.45–1.69)	0.702		
Stage, ≥ II	4.86 (2.13–11.1)	< 0.001	3.82 (1.66–8.81)	0.002
RLNP	0.77 (0.36–1.62)	0.49		
Anastomotic leakage	1.42 (0.65–3.10)	0.36		
Subacute phase pneumonia	1.63 (0.72–3.69)	0.238		
Acute phase pneumonia	2.06 (1.03–4.14)	0.042	1.99 (0.99–4.03)	0.055
Masseter muscle sarcopenia	3.93 (2.09–7.34)	< 0.001	3.10 (1.64–5.86)	0.001
GPS, 1 or 2	1.66 (0.84–3.26)	0.14		

RLNP, recurrent laryngeal nerve paralysis; GPS, Glasgow prognostic score.

### Correlation between L3-PMI and MMI

Supplementary Fig. 1a presents the relationship between L3-PMI and MMI. There was a strong and significant correlation between L3-PMI and MMI (r = 0.53, p = 0.004).

### Receiver operating characteristic curves for predicting postoperative pneumonia

The area under the receiver operating characteristic (ROC) curve was 0.696 for L3-PMI plus MMI for pneumonia (95%CI 0.57–0.81) (Supplementary Fig. 1b). Compared to that for L3-PMI alone and MMI alone, the area under the ROC curve was 0.674 (95%CI 0.56–0.79) for L3-PMI and 0.665 (95%CI 0.43–0.71) for MMI. The areas under the ROC curves for L3-PMI plus MMI were higher than those for L3-PMI alone and MMI alone.

## Discussion

This study showed that preoperative sarcopenia-related masseter muscle loss could be a risk factor for postoperative pneumonia after esophagectomy for esophageal cancer, and that the OS rate may be significantly lower for patients with postoperative pneumonia than for patients without pneumonia. Little is known about the relationship between postoperative pneumonia and masseter muscle loss in the field of gastrointestinal surgery. To the best of our knowledge, this is the first report to demonstrate the effects of MMS on postoperative pneumonia. Postoperative pneumonia following esophagectomy is the most common complication; however, it is known to be the most serious complication along with anastomotic leakage^12^.

In a previous study^1^, the authors retrospectively analyzed 484 cases of curative resection for esophageal cancer, and postoperative pneumonia occurred in 108 (22.3%) patients. Patients with pneumonia were divided into two groups according to the treatment period (within 7 days and after 8 days). Independent risk factors for acute pneumonia were older age, respiratory disease comorbidity, cT3-4 cases, prolonged operation time, and posterior mediastinal reconstruction. DFS was significantly lower in patients with acute pneumonia than in those without pneumonia (p = 0.0002). However, all existing risk factors in their study are perioperative rather than intervenable patient factors.

In contrast, dysphagia is the clinical symptom of swallowing dysfunction. Untreated dysphagia leads to malnutrition, pneumonia, and poor quality of life. The etiology of swallowing dysfunction includes central nervous system disorders, such as those of the cortex, basal and brain stems, and efferent/afferent peripheral nerve dysfunction (motor and sensory or muscle function)^14^. In 2014, Wakabayashi first proposed the concept of sarcopenic dysphagia^15^. The definition of sarcopenic dysphagia is “dysphagia due to sarcopenia in both generalized skeletal muscles and swallowing-related muscles,” and those without sarcopenia in the whole body are excluded^16^.

Sarcopenic dysphagia is a reversible syndrome and an essential concept that can be improved through early interventions. Loss of muscles of swallowing is related to aging and associated with swallowing dysfunction^16^. Tongue thickness evaluated using ultrasonography^17^ and pharyngeal wall thickness measured using magnetic resonance imaging^18^ have been reported as surrogate assessments of swallowing-related muscles; however, both are not routine examinations in gastrointestinal surgery and are difficult to apply in clinical practice. Swallowing consists of the (1) oral phase, (2) pharyngeal phase, and (3) esophageal phase. The masseter muscle is an important skeletal muscle during mastication in the oral phase^16^; the masseter muscle area can be easily evaluated using preoperative CT. Furthermore, the masseter muscles are predominantly composed of type I muscle fibers, also known as slow muscle fibers, which are more strongly atrophied by disuse than by aging^16^. Therefore, we focused on the relationship between masseter muscle loss due to malnutrition or dysphagia before esophagectomy and postoperative pneumonia, which is a common complication of esophagectomy.

In the subgroup analysis of this study, factors such as low FEV_1.0_ and RLNP, which are difficult to intervene, were significantly associated with acute pneumonia. These results were consistent with previous reports^1,4–7^. Interestingly, these factors were not associated with an increased risk of subacute phase pneumonia (respectively: p = 0.363, p = 0.537). Although there was no significant difference, likely owing to a small sample size, MMS tended to be a risk factor not only for acute phase pneumonia but also for subacute phase pneumonia (respectively: p = 0.077, p = 0.078). Subacute phase pneumonia is a serious complication that reduces quality of life, prolongs hospital stay, and adversely affects the continuation of chemotherapy^19^. Improving MMS through preoperative intervention may contribute to reducing postoperative pneumonia for esophageal cancer. In addition, MMS is an independent risk factor for decreased OS, and may contribute not only to pneumonia but also to OS improvement.

In this study, a low L3-PMI alone (sarcopenia group) or masseter muscle loss without sarcopenia was not a risk factor for postoperative pneumonia; however, significant postoperative pneumonia was noted in the MMS group with both low L3-PMI and low MMI. Furthermore, there was a positive correlation between L3-PMI and MMI (r = 0.531, p = 0.004) and the area under the ROC curve was higher for L3-PMI plus MMI than for L3-PMI alone and MMI alone (0.696 vs. 0.674 vs. 0.665, respectively). These results supported the concept of sarcopenic dysphagia proposed by Wakabayashi et al.^16^.

Evaluation of sarcopenic dysphagia using both masseter muscles and psoas muscles might be a useful indicator with higher diagnostic ability in predicting postoperative pneumonia following esophagectomy.

In addition to conventional exercise and nutrition therapy for sarcopenia, postoperative pneumonia following esophagectomy and OS of esophageal cancer can be improved through the following factors: oral care, including denture adjustments or improvement of oral hygiene^20–23^; early chewing and swallowing-related muscle training aimed at recovery of tongue pressure and masseter muscle function. Specifically, several reports suggest that bilateral chewing exercises using a NOSICK exerciser (NOSICK EXERCISER, HIFEELWORLD Inc.) are effective in improving occlusal force and masseter muscle thickness in healthy elderly individuals^24^. The NOSICK exerciser is a U-shaped oral exercise device that was developed for rehabilitation purposes and the performance of masticatory muscle exercises in patients with jaw joint disorders; the elasticity of three small springs inside the device allows resistance during chewing. This device might be effective for masticatory muscle exercises in patients with esophageal cancer.

Our work has some limitations. First, this study was a single-center retrospective study with a small number of patients. Second, the definition of sarcopenia in this study was based on muscle mass alone; however, the European Working Group on Sarcopenia in Older People^25^ recommends that muscle strength or walking speed should also be considered in this diagnosis. Third, the cut-off values for L3-PMI or MMI remain controversial. Specifically, the cut-off values for L3-PMI determined in a large number of Asian adults were used in this study, considering race and sex. Finally, patient nutritional status was only evaluated using the preoperative Glasgow Prognostic Score, while postoperative oral intake, weight loss, or occlusal force were not assessed. There are few clinical studies on masseter muscle sarcopenia, and further large-scale studies including other ethnicities and races are warranted in the future.

In conclusion, preoperative masseter muscle loss due to sarcopenia is significantly associated with postoperative pneumonia in patients who underwent esophagectomy for esophageal cancer. Preoperative MMI in combination with L3-PMI may serve as a useful indicator for predicting the incidence of postoperative pneumonia in patients with esophageal cancer.

## Methods

### Study design

This retrospective cohort study included patients who underwent surgical resection for esophageal cancer at the International University of Health and Welfare Hospital (Nasushiobara, Tochigi Prefecture, Japan) between March 2013 and October 2021. We retrospectively examined the primary endpoint as the occurrence of postoperative pneumonia within 3 months of surgery using a maintained database. The inclusion criteria were thoracoscopic and laparoscopic (including robot-assisted) R0 esophagectomy for esophageal cancer with complete follow-up data and clinical details. The exclusion criteria were palliative surgery (n = 1) or transition to open thoracotomy (n = 2) and perioperative death (n = 1). This study was conducted in accordance with the Declaration of Helsinki and approved by the Institutional Review Board of the International University of Health and Welfare Hospital (approval number: 21-B-461). All the participants were given the opportunity to opt out of their records from being used in these analyses.

### Treatment and patient management

Surgical indications, surgical treatment, chemotherapy selection, and basic surveillance for esophageal cancer were performed according to the standard practice guidelines for esophageal cancer (2017), edited by the Japan Oesophageal Society^26^. Staging and pathological diagnosis were performed according to the 11th edition of the Japanese Classification of Esophageal Cancer^27^. Neoadjuvant chemotherapy was administered in patients with clinical stage II or III cancer, except those with severe stenosis. Neoadjuvant chemotherapy consisted of two cycles of 5-fluorouracil and cisplatin.

### Surgical procedure and perioperative management

A clinical pathway was introduced in all patients during the perioperative management of esophagectomy^28^. At the time of the first visit, the instructions included smoking cessation, preoperative respiratory rehabilitation, and maintenance of oral hygiene. All operations were performed by a single experienced surgeon, who was a licensed attending doctor for laparoscopic surgery. First, thoracoscopic or robot-assisted esophagectomy with mediastinal lymph node dissection was performed in the prone position. After the thoracic procedure, the patients were placed in a supine position, and neck dissection, gastric mobilization with abdominal dissection, and gastric tube or ileocolic conduit reconstruction were performed. Finally, a jejunostomy was performed. Perioperative antibiotics and steroids used included 1 g cefazolin and 1000 mg methylprednisolone administered 30 min before the first incision and an additional dose of cefazolin administered every 3 h intraoperatively.

All patients were admitted to the intensive care unit for routine postoperative mechanical ventilation. Extubation was performed based on the arterial blood gas analysis, chest radiography, and bronchoscopy on postoperative day 1. Early enteral nutrition was initiated within 24 h of surgery. Patients generally started oral intake on the seventh day after surgery, which was gradually increased over time.

### Definition of postoperative complications

Postoperative pneumonia and RLNP were defined as grade II or higher complication according to the Clavien–Dindo classification within 3 months after the surgery. Pneumonia was diagnosed based on increased temperature, leukocyte count, and pulmonary radiography findings^19^, and RLNP was diagnosed using visualization using bronchoscopy. Pneumonia in the acute phase was defined as pneumonia that occurred within 7 days of surgery, while pneumonia in the subacute phase occurred after 8 days^1^.

### Definition of sarcopenia using psoas and masseter muscles (Fig. 2a,b)

**Figure 2:**
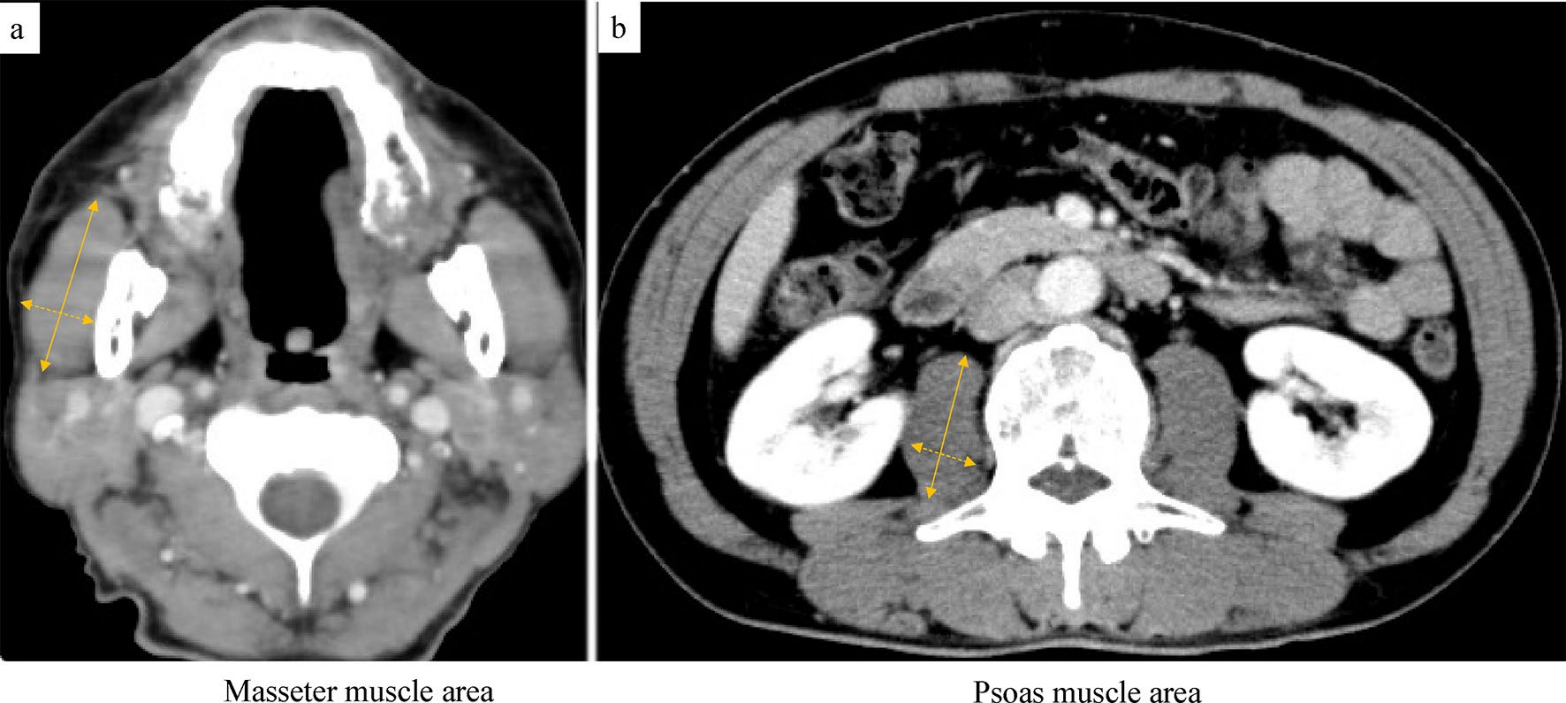
(**a**) The masseter muscle area was calculated 2 cm below the zygomatic arch on the right using the following formula: length of the major axes (continuous line) × the length of the minor axes (dotted line).  (**b**) The psoas muscle area was calculated at L3 on the right using the following formula: length of the major axes (continuous line) × the length of the minor axes (dotted line).

Cervical-to-abdominal enhanced computed tomography (CT) performed within 1 month of the surgery was used to estimate the psoas and masseter muscle areas. The bilateral psoas muscle area was measured at L3 using the following formula: (length of the major axes × length of the minor axes), and L3-psoas muscle index (PMI) was calculated as follows: psoas muscle area/height squared (cm^2^/m^2^)^13^. The sex-specific cutoff values used to define sarcopenia were 6.36 cm^2^/m^2^ for men, and 3.92 cm^2^/m^2^ for women, based on a previous study by Hamaguchi et al.^13^. Sarcopenia was defined as PMI below the sex-specific cutoff values.

The bilateral masseter muscle area was calculated 2 cm below the zygomatic arch in the axial plane using the following formula: (length of the major axes × the length of the minor axes) (Fig. 2b)^29,30^, and the masseter muscle index (MMI) was calculated as follows: masseter muscle area/height squared (cm^2^/m^2^). Given the absence of clearly defined diagnostic thresholds for masseter muscle loss, the sex-specific MMI cutoff values used to define MMS were 3.11 cm^2^/m^2^ for men and 3.29 cm^2^/m^2^ for women, maximizing the Youden index for predicting pneumonia for each sex on the ROC curve.

Masseter muscle sarcopenia (MMS) was defined as (1) MMI below the cutoff values and (2) sarcopenia.

### Statistical analysis

Mann–Whitney U and chi-square tests were used to compare the continuous and dichotomous categorical variables, respectively. Variables with a p-value < 0.05 in the univariate analysis in predicting postoperative pneumonia were analyzed using the multivariate logistic regression model. Spearman’s correlation coefficient was used to evaluate the linear relationship between two continuous variables.

Kaplan–Meier curves with log-rank test were used to estimate and compare the survival between the patients with and those without pneumonia. The Cox proportional hazards model was used to estimate the hazard ratios for overall survival (OS) in univariate and multivariate analyses. The cut-off levels of % vital capacity (%VC) and forced expiratory volume in one second (FEV_1.0_) in univariate and multivariate analyses were 80% and 1.5 L, respectively^31^. A two-sided P-value < 0.05 was considered significant. STATA/IC version 16.0 (STATA Statistical Software; StataCorp, College Station, TX, USA) was used for statistical analysis.

### Human rights statement and informed consent

All procedures followed for the conduction of this study were in accordance with the ethical standards of the responsible committee on human experimentation (institutional and national) and with the Helsinki Declaration of 1964 and later versions. The study was approved by the Institutional Review Board of the International University of Health and Welfare Hospital (approval no: 21-B-461). All data were subject to strict privacy policies, and the patients or their family members had the option to drop out of the study at any time. The requirement for informed consent acquisition from the patients was waived because of the retrospective design of this study and anonymized data by the Institutional Review Board of the International University of Health and Welfare Hospital.

## Supplementary Information

Supplementary Figure 1.

## Data Availability

The data that support the findings of this study are available from the corresponding author upon reasonable request.
