# Supplementary material for: Association between masseter muscle sarcopenia and postoperative pneumonia in patients with esophageal cancer
Source: Sci Rep. 2022 Sep 30;12:16374. doi: 10.1038/s41598-022-20967-1 (PMC9525668; doi:10.1038/s41598-022-20967-1)
Supplement: Supplementary file 1 — Supplementary Figure 1. [file 41598_2022_20967_MOESM1_ESM.pdf]

(a)

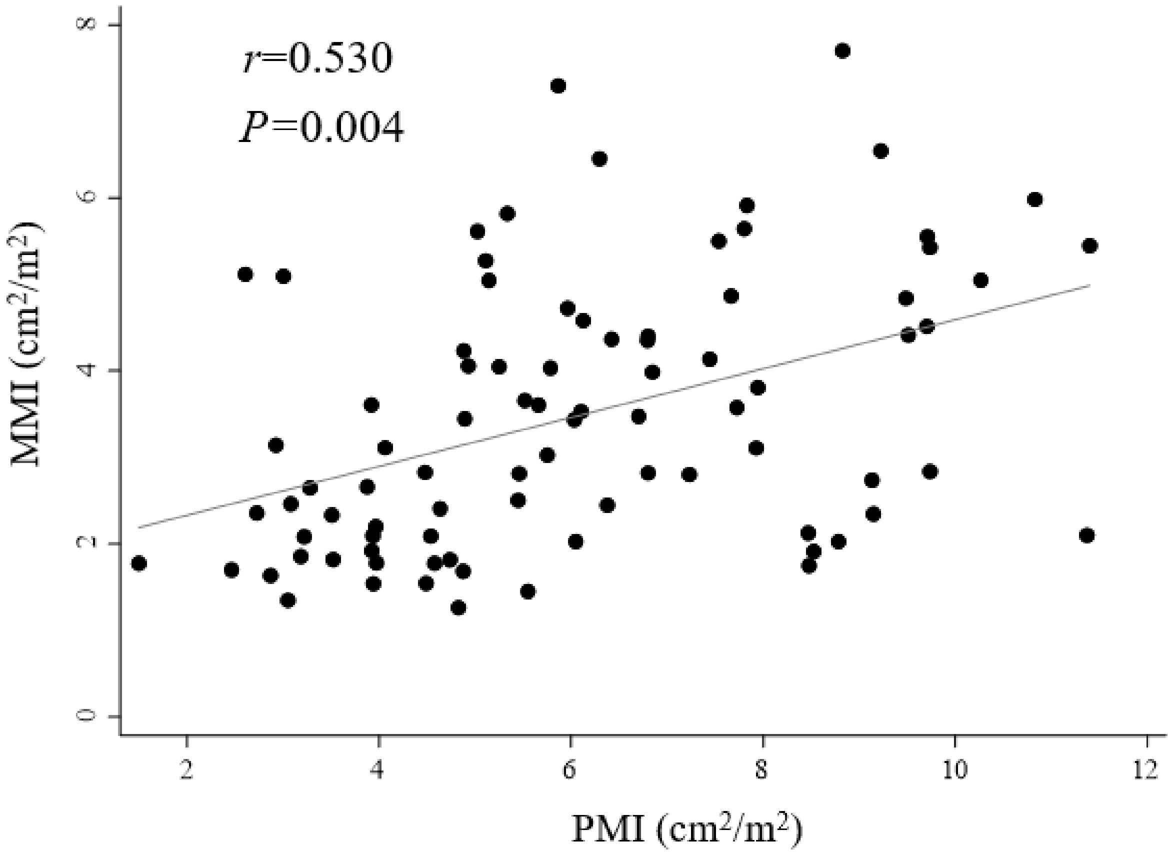

(b)

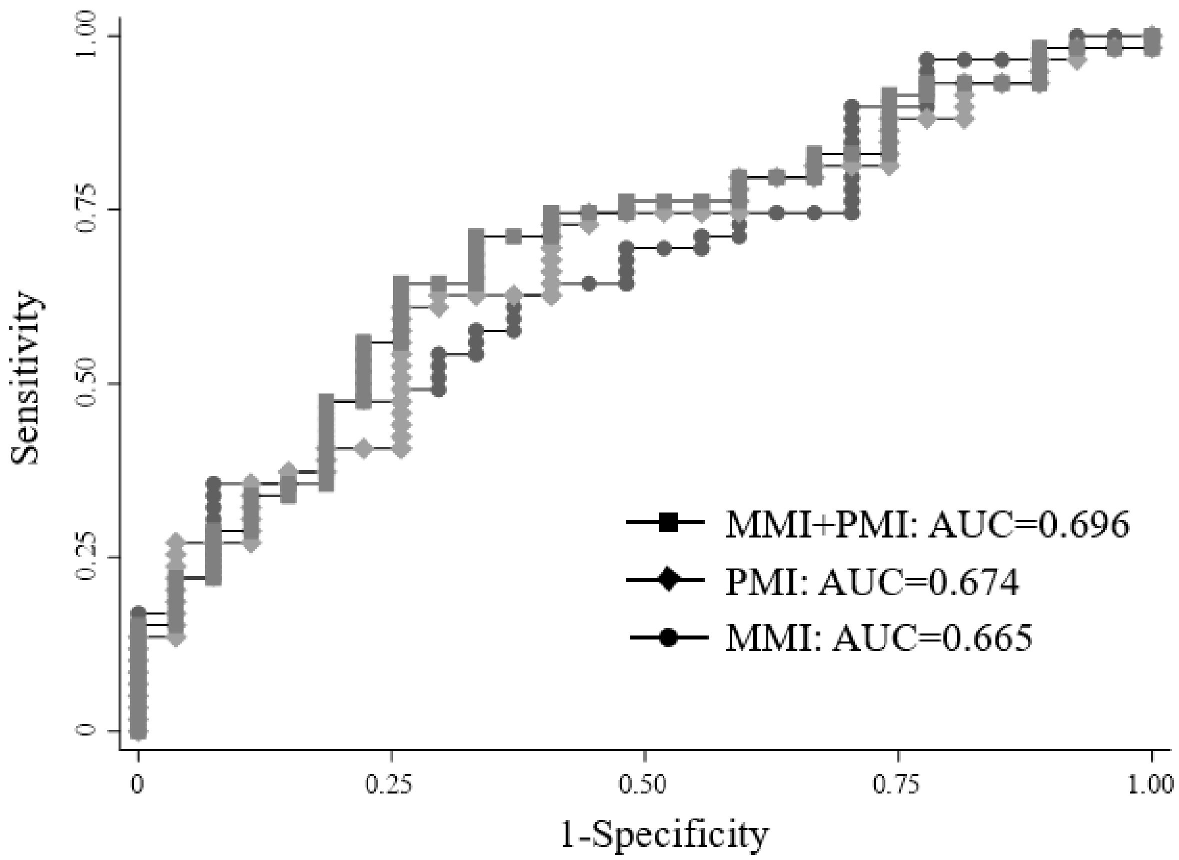

(a) Masseter muscle index strongly and significantly correlated with L3-skeletal muscle index.

(b) Receiver operating characteristic curves for the prediction of postoperative pneumonia in patients with esophageal cancer (masseter muscle index plus L3-skeletal muscle index vs. L3-skeletal muscle index alone vs. masseter muscle index alone).
